# Physical mapping and BAC-end sequence analysis provide initial insights into the flax (*Linum usitatissimum *L.) genome

**DOI:** 10.1186/1471-2164-12-217

**Published:** 2011-05-09

**Authors:** Raja Ragupathy, Rajkumar Rathinavelu, Sylvie Cloutier

**Affiliations:** 1 Cereal Research Centre, Agriculture and Agri-Food Canada, 195 Dafoe Rd, Winnipeg, MB, R3T 2M9, Canada; 2 Genomics & Bioinformatics Division, ITC Research & Development Centre, Bangalore, India; 3 Department of Plant Science, University of Manitoba, 66 Dafoe Rd, Winnipeg, MB, R3T 2N2, Canada

## Abstract

**Background:**

Flax (*Linum usitatissimum *L.) is an important source of oil rich in omega-3 fatty acids, which have proven health benefits and utility as an industrial raw material. Flax seeds also contain lignans which are associated with reducing the risk of certain types of cancer. Its bast fibres have broad industrial applications. However, genomic tools needed for molecular breeding were non existent. Hence a project, Total Utilization Flax GENomics (TUFGEN) was initiated. We report here the first genome-wide physical map of flax and the generation and analysis of BAC-end sequences (BES) from 43,776 clones, providing initial insights into the genome.

**Results:**

The physical map consists of 416 contigs spanning ~368 Mb, assembled from 32,025 fingerprints, representing roughly 54.5% to 99.4% of the estimated haploid genome (370-675 Mb). The N50 size of the contigs was estimated to be ~1,494 kb. The longest contig was ~5,562 kb comprising 437 clones. There were 96 contigs containing more than 100 clones. Approximately 54.6 Mb representing 8-14.8% of the genome was obtained from 80,337 BES. Annotation revealed that a large part of the genome consists of ribosomal DNA (~13.8%), followed by known transposable elements at 6.1%. Furthermore, ~7.4% of sequence was identified to harbour novel repeat elements. Homology searches against flax-ESTs and NCBI-ESTs suggested that ~5.6% of the transcriptome is unique to flax. A total of 4064 putative genomic SSRs were identified and are being developed as novel markers for their use in molecular breeding.

**Conclusion:**

The first genome-wide physical map of flax constructed with BAC clones provides a framework for accessing target loci with economic importance for marker development and positional cloning. Analysis of the BES has provided insights into the uniqueness of the flax genome. Compared to other plant genomes, the proportion of rDNA was found to be very high whereas the proportion of known transposable elements was low. The SSRs identified from BES will be valuable in saturating existing linkage maps and for anchoring physical and genetic maps. The physical map and paired-end reads from BAC clones will also serve as scaffolds to build and validate the whole genome shotgun assembly.

## Background

Flax (*Linum usitatissimum *L.) was domesticated for its seed oil and stem fibres nearly 7,000 years ago, during the Neolithic period [1]. However, recently discovered 30,000 year old flax fibres from the upper Paleolithic period suggest that flax was used by humans prior to its domestication [2]. Today, flax is grown as an oilseed (linseed) crop or a fibre crop. Linseed oil, rich in the omega-3 fatty acid (alpha linolenic acid), is being used in the fabrication of biodegradable ('green') products such as linoleum besides gaining acceptance as a functional food with numerous proven health benefits [3]. Flax bast fibres are well known as linen but have a variety of other applications. Therefore, flax is not only a historically important species; it remains an important, versatile and expanding crop today, in many parts of the world. Until recently, flax improvement relied mostly on conventional breeding methods often limited to an adapted but narrow germplasm base. Genomics resources such as BAC libraries, genetic and physical maps, QTL analysis, BES and whole genome sequence are emerging, promising to enhance breeding processes. In 2009, the Total Utilization Flax GENomics (TUFGEN; http://www.tufgen.ca) project was initiated in Canada to generate genomics resources for flax and to develop a comprehensive knowledge of its unique genome with specific goals in applied genomics aiming at the improvement of flax as a total utilization crop.

Flax belongs to the family Linaceae, order Malpighiales. The genus *Linum *consists of approximately 200 species, of which *L. angustifolium Huds*. is considered the wild progenitor of cultivated flax, *Linum usitatissimum L*. The latter is a self pollinated diploid species with a chromosome number of 2n = 30 [4]. The flax genome was estimated to contain a C-value of 0.7 pg, equivalent to ~675 Mb in size ([5]; http://data.kew.org/cvalues/) and to have unique characteristics [6]. However, a recent estimate of the size of the CDC Bethune flax genome of 0.38 pg/C would translate into only 370 Mb (Michael Deyholos and David Galbraith, personal communication). Environmental induction of stable heritable changes resulting in extreme differences for plant weight, height and DNA content were reported in the flax lines Stormont Cirrus, Rembrandt, Hollandia and Liral Monarch where they were referred as large stable genotrophs (L1) and small stable genotrophs (S1) [6,7]. Flax nuclear DNA with ~35% highly repetitive tandemly arrayed sequences, ~15% middle repetitive fraction and ~50% low-copy number fraction is reported to be somewhat different from other plant genomes characterized to date [7]. Classical cytogenetic studies identified two nucleolar organizer regions (NOR) harbouring rDNA loci with tandem arrays of repeating units of 8.6 kb in length, encoding 45S rRNA transcriptional units and spacer DNA and serving as precursors of 25S, 5.8S and 18S rRNAs [8]. In contrast, 5S rRNA loci were distributed over many chromosomes of flax as tandem arrays of 350-370 bp, consisting of a 120 bp transcription unit and a 230 bp spacer DNA [9,10]. Nearly 3% of the flax genome was estimated to represent the 5S rRNA multigene family with ~117,000 copies per diploid genome [10], compared to 0.7% in *Arabidopsis *[11]. Also, 5S rDNA multigene family members were found to have several classes which were more heterogeneous than 45S rDNA, in terms of sequence divergence [9]. A high-density microarray platform was recently developed which is suitable for analyzing differential gene expression of biologically relevant samples [12].

Large insert genomic libraries constructed with bacterial artificial chromosomes (BAC) are known for their high degree of genomic insert structural stability and easy handling of *E. coli *host cells. BAC libraries are useful in generating physical maps, in sequencing using a clone by clone based sequencing strategy and minimum tiling paths and in map-based cloning of agronomically important genes such as disease resistance genes.

A physical map represents a genomic region (single locus) or an entire genome, constructed by set(s) of overlapping large-insert clones in which the distances are measured in base pairs [13]. Contigs are built following the analysis of a large number of BAC clone fingerprints obtained by size determination after digestion with a number of restriction enzymes [14]. Clone overlap is determined by statistical analysis, employing the FPC algorithm [15]. BAC-based whole genome physical maps have been constructed in rice [16,17], *Arabidopsis *[18,19], maize [20], soybean [21], bean [22], *Brassica rapa *[23], *Brachypodium *[24], papaya [25] and melon [26].

BAC-end sequencing refers to the bidirectional end sequencing of the genomic DNA insert with the help of universal priming sites in the cloning vector. They were proposed as sequence tagged connectors (STC) for generating accurate assembly of the whole genome shotgun sequence of the human genome [27] because of the constraints imposed in the assembly in terms of distance and orientation between mate-pairs. At an optimal redundancy level of coverage required in genome projects, whole genome BES cover ~5-10% of the genome and, as such, their annotation can provide initial insights into the composition of a genome as reported in rice [28], maize [29], Korean ginseng [30], papaya [31], *Brassica rapa *[32], wheat 3B [33], *Musa acuminata *[34], white clover [35], *Brachypodium *[36], potato [37], tomato [37], citrus [38], apple [39] and carrot [40]. They are also a good source of genomic simple sequence repeats (SSRs) which serve as reliable landmarks across the genome upon genetic mapping, as reported in plant genomes such as cotton [41] and *Brassica napus *[42]. Also, BES are useful in anchoring the physical and genetic maps as reported in rice [16] and soybean [43].

Gene ontology (GO) provides a set of unified and structured vocabularies that describe gene products and their annotations in the context of cellular components where they are localized, biological processes in which they are involved and molecular functions they perform, thereby classifying them into functional categories independent of organisms ([44]; http://www.geneontology.org). For instance, in castor bean, a phylogenetically related taxa of flax, 43,657 GO terms were assigned to 14,991 proteins [45]. Similarly, GO annotations of 59,626 EST derived flax unigenes suggested 16.8%, 24.3% and 27.8% of sequences could be assigned to molecular functions, biological processes and cellular components, respectively [12]. Further categorization of gene annotations on the basis of a relatively small set of high-level GO terms, called GO-slim categories, provides a broad overview of biology encoded by the genome [44] indicating its uniqueness.

In this study, we report the generation of a whole genome physical map of flax, and sequencing and annotation of 80,337 BAC-ends, providing initial insights into the content and composition of the flax genome.

## Methods

### BAC libraries

BAC libraries of the cultivar CDC Bethune were constructed from high molecular weight DNA isolated from 10 g of young leaf tissue by BIO S&T Inc. (Montreal, Canada). Two restriction enzymes, *HindIII *and *BamHI*, were used for partial digestion of mega-size DNA, cloned in the pIndigoBAC-5 vector (Epicentre Inc., Madison, USA) and transformed in the *E. coli *strain DH10B (Invitrogen, Canada). LB medium containing 12.5 μg/ml chloramphenicol, 50 μg/ml X-Gal and 25 μg/ml IPTG ensured selection of recombinant clones based on the insertional inactivation of the *lac Z *gene prior to arraying in 384 well plates. The *HindIII *library consists of 40,704 clones with an average insert size of 150 kb and the *BamHI *library consists of 51,456 clones with an average insert size of 135 kb. Genome coverage of ~8.7X and ~9.9X were estimated for the *HindIII *and *BamHI *libraries, respectively, based on the 675 Mb original size estimate of the flax genome.

### BAC fingerprinting and physical mapping

A total of 43,776 CDC Bethune BAC clones (comprising 20,352 and 23,424 from the *HindIII *and *BamHI *libraries, respectively) were used for agarose gel based fingerprinting and assembly after double digestion with *EcoRI *and *EcoRV*, following the protocol of Mathewson et al [46], at the Genome Sciences Centre of the British Columbia (BC) Cancer Agency. Fingerprints representing potential repetitive regions and cross well contaminations were filtered out using an in-house automated gel analysis software called 'ClipLanes'. Prior to assembly, a procedure called 'mapmopping' was performed on the fingerprints to further filter out clones that contained more than 135 restriction fragments, and then those that had an insert over 260 kb in size and in excess of 110 fragments. The resultant high quality fingerprints were assembled using the FPC algorithm (Fingerprinting contigs, [15]), initially using a high stringency cutoff value of 1e^-16 ^and a tolerance of 7. If there were shared marker fragments, the cutoff was altered depending on the number of shared markers, as follows: one marker, 1e^-15^; two markers, 1e^-14^; three markers, 1e^-13^. A series of six automated contig merging rounds were then performed at the fixed tolerance of 7, each round dropping slightly in stringency of overlap expected between end clones: 1) 1e^-14 ^cutoff, requiring two end clones; 2) 1e^-12 ^cutoff, requiring two end clones; 3) 1e^-10 ^cutoff, requiring two end clones; 4) 1e^-14 ^cutoff, requiring one end clone; 5) 1e^-12 ^cutoff, requiring one end clone and 6) 1e^-10 ^cutoff, requiring one end clone. A total of 129 EST-SSR markers were initially used to anchor the FPC contigs to a genetic map [47] as well as to validate the assembly.

### BAC-end sequencing

Bidirectional-end sequencing of the 43,776 fingerprinted BAC clones was also carried out at the Genome Sciences Centre by the standard Sanger dideoxy chain termination method using Big-Dye v3.1 chemistry and an ABI 3730 or 3730XL DNA Analyzer (Applied Biosystems, CA, USA). Base calling was carried out using PHRED [48] and the resultant sequences were processed by removing reads of less than 80 bp in length.

### Identification of known repeats

Processed BES were analysed with the Repeatmasker v-3.2.8 pipeline of the Institute of Systems Biology http://www.repeatmasker.org for identifying known classes of repeats using the Repbase update database (db), subset *Viridiplantae *[49]. Independent homology searches (BLASTn) of BES against the TIGR plant repeat database [50] was also carried out to generate additional evidence of known repeat contents, especially rDNA content.

### Identification of unique flax repeats

Self-BLASTn (E-value threshold of e^-25^) was performed using repeat masked BES (80,337 sequences in total) to identify sequences that had strong matches to multiple sequences in the BES dataset, representing potential novel uncharacterized repeat sequences from the flax genome not available in the public domain. Queries with a minimum of ten hits over minimum thresholds of 80 bp length and 80% identity were extracted and clustered to form mutually exclusive groups. For individual groups, consensus sequences (contigs) were generated by assembly using CAP3 [51]. The reads not assembling into consensus sequences were termed singletons even though they represented more than nine BES. The putative novel flax repeats identified were queried by homology searches (BLASTn) against a number of databases, namely Repbase, TIGR plant repeats, flax-EST, NCBI-EST and NCBI-nt with an E-value threshold of 10^-2 ^to probe their unique nature.

### Simple sequence repeats (SSRs)

Simple sequence repeats were mined from the BES using the algorithm MISA [52] with criteria of a minimum six repeats for dinucleotide motifs and five repeats for trinucleotide and tetranucleotide motifs. For comparative analysis, SSRs were also mined from whole genome assemblies of castor bean, poplar, grapevine, soybean, cucumber, *Arabidopsis*, papaya, rice, sorghum, *Brachypodium *and maize publicly available at http://www.phytozome.net (v6) and apple genome sequence available at ww.rosaceae.org.

### Identification of coding regions

BES masked for previously characterized repeats were used for BLASTn homology searches against an in-house EST db comprising 243,272 ESTs from flax and the NCBI-EST db. BLASTx homology searches of BES against the non-redundant (nr) protein db of NCBI were also carried out. The number of hits was limited with an E-value cut off of e^-5 ^or lower, as previously described [40].

Using the BioPerl toolkit [53], parsing of BLASTn results was done by applying a filter of a minimum of 80% identity over a minimum length of 80 bp. Parsing of BLASTx results was done with the criteria of an alignment length of at least 34 amino acids [31] and a minimum of 35% identity, as suggested by the Gene Ontology Consortium (http://www.geneontology.org/GO.annotation.SOP.shtml).

### Gene ontology

Gene ontology (GO) annotations ([54]; http://www.geneontology.org) were obtained from the results of BLASTx analysis by mapping GI numbers of the NCBI-nr protein db to the existing annotations of characterized proteins in the UniProtKB db [55]. Plant GO-slims for all three independent GO categories namely, cellular components, molecular functions and biological processes were obtained from all GO terms associated with the BLASTx gene annotation list by using the GO slim viewer from the AgBase web server ([56]; http://www.agbase.msstate.edu).

## Results

### Physical mapping

**Figure 1:**
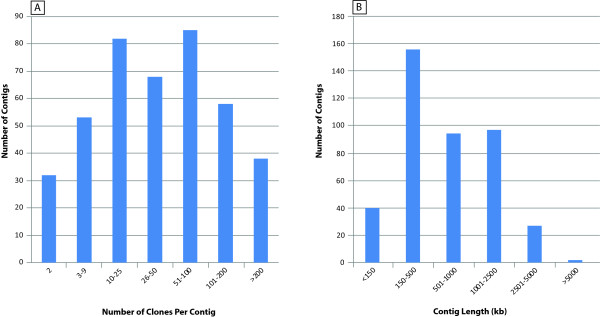
**Physical map of the flax genome (*Linum usitatissimum *cv CDC Bethune):** A. Distribution of the number of clones per contig; B. Distribution of the contigs length.

From a total of 43,776 CDC Bethune BAC clones, fingerprint data was collected on 456 agarose gels, from which 35,585 clones (81.2%) were identified to have fingerprints suitable for downstream processing and contig building. Further stringent filtering for high quality by excluding fingerprints representing clones with repetitive regions of the genome, resulted in 32,025 fingerprints. In addition, 167 clones were excluded from contig building by the mapmopping procedure to remove clones with very large inserts and many bands. Finally, 31,858 fingerprints were taken up for contig assembly. The initial physical map consisted of 1,096 contigs and 2,035 singletons and a series of six automated contig merging rounds (see methods) resulted in 417 final contigs. Among them, contig 1,122 with 796 clones (of which 702 were buried) was identified to represent the flax chloroplast genome and was therefore removed from the physical map. The summary of the flax physical map is presented in Table 1. The final physical map consists of 416 contigs spanning 157, 213 consensus band (CB) units from 29,027 clones (Additional file 1: Table S1). A total of 96 contigs contain more than 100 clones and 32 contigs contain only two clones (Figure 1A). The total physical length of all contigs, which is calculated using the average fragment (band) size of the clone fingerprints (2,342 bp) and number of fragments across all contigs (157,213) was estimated to span 368,192,846 bp (~368 Mb). The contigs range in size from ~5,562 kb (contig # 21; 437 clones; 2375 CB units) to ~32.8 kb (contig #1092; 2 clones; 14 CB units) (Additional file 1: Table S1). There are 126 contigs more than 1,000 kb in size (Figure 1B).

**Table 1:** Summary of flax (*Linum usitatissimum *L.) cv CDC Bethune physical map

Description	Total
Number of BAC clones fingerprinted	43,776
Number of high quality fingerprints used for assembly	32,025
Average number of valid bands per clone	64
Number of contigs	416
Number of singletons	2,035
Total length of the contigs	368,192,846 bp
N50 contig length	1,494 kb
Longest contig	5,562 kb
Average number of clones per contig	71
Number of genetic markers used for anchoring contigs	129

### Anchoring the contigs to the genetic map

A total of 96 contigs were anchored to 129 EST-SSR markers which were genetically mapped, allowing positioning of physical contigs onto the genetic map. Of these, 56 contigs were anchored with one marker each, 30 contigs with two, five contigs with three, three contigs with four markers and two contigs were anchored with five markers each. In 80 cases, more than one BAC clone identified with a specific marker or set of markers assembled in a single contig, indicating the accuracy of the assembly. However, 18 markers were positioned in more than one contig with the distribution as follows: 14 markers were present in 2 independent contigs; 3 markers were present in 3 contigs and one marker was present in 4 contigs.

### BAC-end sequencing

Of the possible 87,552 BES from 43,776 BAC clones, 4120 (4.7%) failed to yield any sequence, 145 BES (0.2%) were empty vectors, 1705 BES (1.9%) were shorter than 80 bp and 81,582 BES (93.2%) were of good quality. A total of 1245 reads (1.5%) were found to be similar to the chloroplast genome of *Vitis *and therefore removed from the dataset. Finally, 80,337 BES (~8%-14.8% of the estimated 370-675 Mb flax genome), averaging 679 bp and constituting ~54.6 Mb of sequence data were selected for further analysis. The GC content was estimated at 43.35% (Table 2). The 80,337 BES were deposited at the GSS section of GenBank with accession numbers HR714444-HR752254 representing the 37,811 sequences from the *HindIII *library and HR752255-HR794780 representing the 42,526 sequences from the *BamHI *library.

**Table 2:** Descriptive statistics about BES from flax cv CDC Bethune

Description	Total
Number of BES reads greater than 80 bp in length	81,582
Number of reads with similarity to *Vitis vinifera *chloroplast DNA	1,245
Number of BAC-end sequences without chloroplast DNA	80,337
Total length of chloroplast-free BES	54,600,041 bp
Read average length	679 bp
GC content	43.35

### Characterization of known repetitive sequences

Repeatmasker analysis indicated that 22,958 reads (28.5%) were found to harbour repetitive regions of more than 80 bp in length, among which 6,633 reads (8.2%) were completely masked as repetitive regions. A total of 49,148 reads (61%) did not have any homology to known repetitive sequences of the *Viridiplantae *subset of the Repbase database. A total of 13,228 reads (16.4%) contained rDNA sequences. LTR retroelements were found to be present in 10,213 reads (12.7%) and non LTR retrotransposons and DNA transposable elements were identified in 2,215 reads (2.7%).

In terms of sequence length, the composition of known repeats characterized in BES is summarized in Table 3. In total, ~20.5% of the sequences represent known interspersed repeat elements. The most important component is ribosomal DNA (rDNA) with ~13.8% of total BES, followed by retroelements at 5.8%. In the latter category, long terminal repeat (LTR) elements are predominant with 5.2%, of which LTR-*copia *and LTR-*gypsy *elements composed 3.4% and 1.8%, respectively. In total 62 families of characterized transposable elements were identified (Table 4).

**Table 3:** Composition of known *Viridiplantae *repeats in BES using RepeatMasker

Repeat component	Class	Order	Superfamily	Total no. of elements/units	Total length (bp)	Total length as % in BAC-End sequences
Mobile genetic elements	I. Retroelements			10,576	3,162,436	5.8
		SINE	-	2	89	0.0
		LINE	-	1,176	234,602	0.4
		LTR		9,245	2,900,613	5.3
			*Copia*	4,867	1,850,625	3.4
			*Gypsy/DIRS1*	3,372	985,038	1.8
			Unclassified	1006	64950	0.1
		PLE	Penelope	153	27,132	0.0
	II. DNA transposons			1,094	201,075	0.4
		-	hobo-Activator	371	87,631	0.2
		TIR	Tc1-IS630-Pogo	11	2,036	0.0
		-	En-Spm	249	47,547	0.1
		TIR	MuDR-IS905	250	31,936	0.1
		TIR	Tourist/Harbinger	49	11,187	0.0
		-	Other (Mirage, P-element)	1	49	0.0
		Unclassified	-	163	20,689	0.0
rDNA				13,342	7,516,095	13.8
Satellites				22	1,972	0.0
Simple sequence repeats (SSRs)				2,556	95,533	0.2
Low complexity regions (Homopolymers)				8701	340,090	0.6
Overall length of sequences masked				36291	11317201	20.7

**Table 4:** Families of known mobile genetic elements identified in flax BES

Type	Super Family	No. of Families	Families
Retrotransposon	*Copia*	19	Alfare2, Angela1, Barbara, BARE-2, BNR1, CPSC4A, Maximus, Opie2, Prem3, Shacop11, SPRT1, Stonor, TLC1, TNT1, TONT2, Topscotch, TORTL1, TOS17, TOTO1
	*Gypsy*	27	Atlantys, Bagy1, Bnintmo, Calypshan2, Carep, Cereba, Cinful1, CRM-I, Daniela, Dea1, Del, Diaspora, Erika1, Fatima, Ogre, Grande1, Gret1, Gycume1, Gypot1, Gypshan2, Gypsode1, Megy, Ophelia1, Ram12, Sore1, Tekay, Truncator
	LINEs	5	BALN1, BVL1, CIN4, FMLN1, Shaline10
	SINEs	4	BoSB10A, Casine, Ormosia, Sadhu4-2
DNA Transposon		7	THRIA, TLP3, TNAT1A, TNR1, Tourist, TPN1, TWIF
	Total	62	

Only 14 transposable elements have been partially characterized to date in flax and the results of homology searches of BES against these transposable elements are summarized in Table 5. Among them, the partial sequence of the LTR retroelement FL4 was found to be present in 365 reads, indicating a potential high copy retroelement in the flax genome.

**Table 5:** Known flax transposable elements identified in flax BES

Name of the element	GenBank ID	Length (bp)	Number of hits
Retrotransposons			
FL1a*	GU735098.1	1329	5
FL1b*	GU735096.1	1327	11
FL2*	GU735097.1	318	None
FL4*	GU929874.1	693	365
FL5*	GU929875.1	979	36
FL6*	GU929876.1	800	86
FL7*	GU929877.1	598	74
FL8*	GU929878.1	672	6
FL9*	GU980587.1	468	None
FL10*	GU980588.1	1052	4
FL11*	GU980589.1	1300	None
FL12*	GU980590.1	854	67
Cassandra	DQ767972.1	632	14
DNA transposons			
dLUTE	AF036935.1	314	None

*partial element

Independent homology searches (BLASTn) of the flax BES against the TIGR plant repeat database also identified 13,746 reads (17.1%) as having homology to ribosomal RNA genes, with a total high scoring portion (HSP) length of 7,374,546 bp (~7.3 Mb), resulting in an estimate of 13.5% as the rDNA component of BES.

### Characterization of unique flax repetitive sequences

Self-BLASTn analysis of BES identified 14,475 reads (18.0%) having a coverage of ≥10x with mutually inclusive hits and averaging 279 bp (HSP length). Assembly of these repetitive reads resulted in 456 contigs and 873 singletons, representing the potential novel repeat regions of the flax genome. Singletons in this instance refer to reads harbouring internal repetitive sequences and which were found to have a minimum of 10 hits in the BES dataset. Repeatmasker analysis of all the contigs and singletons (1,329 in total) against Repbase known repeat database identified and masked only 1529 bp (0.13%) of the total length of 1,172,838 bp. Results of homology searches against databases such as TIGR repeats, TREP, flax-EST, NCBI-EST, NCBI-nt and NCBI-nr are summarized in Table 6. A total of 871 sequences were found to not have any hits against the NCBI-nt database, indicating potential novel repeat sequences from the flax genome.

**Table 6:** Summary of homology searches of contigs and singletons representing highly repetitive sequences of flax

	Hits of >80 bp in length			
			
Database	Number of hits	Actual high scoring portion (HSP) (bp)	Number of hits (<80 bp in length)	Number of reads not finding any hits
Repbase-*Viridiplantae*	1	314	135	1193
TIGR repeats	0	-	-	1329
TREP repeats	0	-	5	1324
Flax-EST	498	149,059	222	609
NCBI-EST	231	60,130	185	913
NCBI-nt	385	115,237	73	871
NCBI-nr	261	-	110	958

### Simple sequence repeats

Mining for the presence of simple sequence repeat (SSR) domains identified 4,064 putative SSRs from 3,629 reads. A total of 373 sequences contained more than one SSR and 219 SSRs were present in compound forms. Types and distribution of SSRs are presented in Table 7. In summary, flax SSRs are nearly all trinucleotide (2184 in total; 53.7%) and dinucleotide (1571 in total; 38.7%) motifs. SSRs with tetranucleotide motifs comprise only 4.5%. Motif (AT/AT)_n _was the most abundant (10.6%) followed by (TA/TA)_n _(9.5%), (AG/CT)_n _(8.7%), (GAA/TTC)n (7.5%), (GA/TC)_n _(6.3%), (AGA/TCT)_n _(5.1%) and (AAG/CTT)_n _(4.6%).

**Table 7:** Types and distribution of SSRs in flax BAC-End sequences

	Number of repeats												
	
Motif	4	5	6	7	8	9	10	11	12	13	14	15+	Total
Dinucleotide													
AC/GT	-	-	35	13	13	1	-	1	-	-	-	1	64
CA/TG	-	-	39	25	12	2	3	-	-	-	-	-	81
GA/TC	-	-	92	47	35	25	13	16	7	4	2	15	256
AG/CT	-	-	102	68	51	39	23	16	13	7	6	28	353
TA	-	-	99	71	53	48	32	19	15	13	5	33	388
AT	-	-	112	78	41	34	35	27	21	15	10	56	429
Trinucleotide													
ACG/CGT	-	7	1	-	-	-	-	-	-	-	-	-	8
CGA/TCG	-	5	6	2	-	-	-	-	-	-	-	-	13
CGC/GCG	-	12	2	-	1	-	-	-	-	-	-	-	15
GAC/GTC	-	9	4	3	-	-	-	-	-	-	-	-	16
GTA/TAC	-	6	5	3	2	1	-	-	-	-	-	-	17
GCC/GGC	-	15	2	2	-	-	-	-	-	-	-	-	19
CTA/TAG	-	9	9	2	-	-	-	-	-	-	-	-	20
CAC/GTG	-	19	2	-	-	-	-	-	-	-	-	-	21
ACT/AGT	-	7	12	6	1	-	1	-	-	-	-	-	27
CCG/CGG	-	23	6	3	1	-	-	-	-	-	-	-	33
ACA/TGT	-	19	8	1	6	1	-	-	2	-	-	-	37
CCA/TGG	-	26	11	3	-	2	1	-	-	-	-	-	43
AAC/GTT	-	35	6	3	1	-	1	-	-	-	-	-	46
ACC/GGT	-	26	18	5	-	-	-	-	-	-	-	-	49
AGG/CCT	-	35	12	7	-	-	-	-	-	-	1	-	55
GCA/TGC	-	45	6	5	4	-	-	-	-	1	-	-	61
CTC/GAG	-	41	6	10	4	1	-	-	-	-	-	-	62
CAA/TTG	-	31	20	3	4	-	3	1	1	-	-	-	63
CAG/CTG	-	36	13	8	6	1	2	1	-	2	-	-	69
AGC/GCT	-	50	17	2	2	-	1	-	-	-	-	-	72
ATG/CAT	-	48	22	8	2	3	1	-	-	-	-	-	84
TAA/TTA	-	32	25	12	16	2	2	-	2	2	-	1	94
GGA/TCC	-	56	27	9	5	-	2	-	-	-	-	-	99
TCA/TGA	-	50	32	14	5	-	1	1	-	-	-	-	103
ATA/TAT	-	51	14	9	10	6	6	5	4	1	-	2	108
AAT/ATT	-	56	22	19	11	5	6	-	4	2	1	-	126
ATC/GAT	-	67	37	13	5	5	-	-	-	-	-	-	127
AAG/CTT	-	80	46	27	11	4	5	6	2	-	-	4	185
AGA/TCT	-	96	51	20	16	8	4	5	2	1	-	3	206
GAA/TTC	-	162	63	31	21	11	5	8	2	1	-	2	306
Tetranucleotide	-	118	36	12	5	3	7	-	-	-	-	-	181
Other higher order motifs	71	45	4	7	-	1	-	-	-	-	-	-	128

**Total**	71	1317	1024	551	344	203	154	106	75	49	25	145	4064

**Figure 2:**
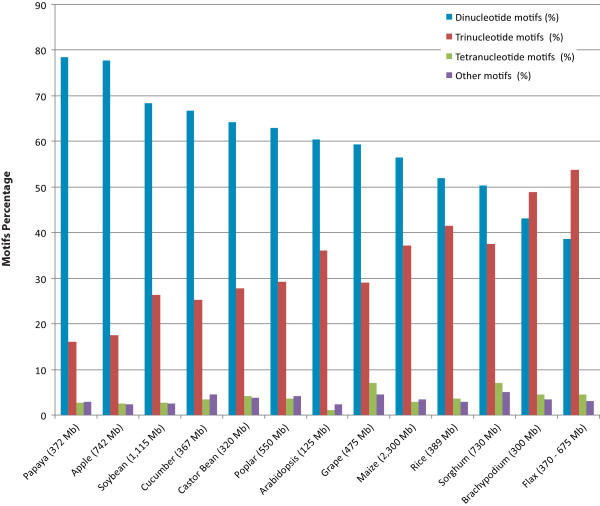
**The SSR motif distribution in sequenced plant genomes in comparison with the BES-based estimates in flax**. For comparative analysis, SSRs were also mined from whole genome assemblies of castor bean, poplar, grapevine, soybean, cucumber, *Arabidopsis*, papaya, rice, sorghum, *Brachypodium *and maize publicly available at http://www.phytozome.net (v6) and apple genome sequence available at ww.rosaceae.org. As per the data citation policy of phytozome, individual references are listed in Additional file 2: Table S2.

Comparison of SSRs among 12 publicly available plant genomes showed that the proportion of dinucleotide and trinucleotide repeats vary greatly (Figure 2; Additional file 2: Table S2).

### Characterization of coding regions

A summary of all BLAST analyses of the BES against the in-house flax-EST, NCBI-EST and NCBI-nr protein databases is presented in Table 8. A total of 21,532 reads (26.8%) were found to be homologous to flax-ESTs, with a cumulative length of hits participating in the alignment accounting for ~9.7% of the total BES dataset of ~54.6 Mb. However, homology searches against the NCBI-ESTs found hits for only 17,038 reads (21.2%), with HSP accounting for ~6.1% of the total BES data. BLASTx searches against the NCBI-nr protein database identified 24,962 reads (~31.1%; e^-5 ^as cut-off) and 14,288 reads (~17.8%; e^-25 ^as cut-off) (Additional file 3: Table S3). A total of 6,637 reads (~8.3%) were predicted to harbour coding regions based on evidence from both EST and protein hits.

**Table 8:** Summary of BLAST analyses of BAC-End sequences of flax (*Linum usitatissimum *cv CDC Bethune)

S. No	Database	No of BAC-End reads harbouring regions of similarity		No of hits as proportion of total **number of BAC-End reads**^**@ **^**(%)**	Total HSP score	Proportion as % of total length of BAC-End-sequences*
		cutoff e^-5^	cutoff e^-25^			
1	Flax-EST	21,532	-	26.8	5,303,617	9.7
2	NCBI-EST	17,038	-	21.2	3,349,832	6.1
3	NCBI-Protein-nr	24,962	14,288	31.1 (e^-5^) 17.8 (e^-25^)	-	-

@Total number of BAC-end reads: 80,337

* Total length of BAC-End sequences: 54,600,041 bp

In depth categorization of translated alignments suggested only ~1% of hits as *Linum*-related proteins with a high similarity of predicted flax proteins to primarily unknown/hypothetical proteins from castor bean (33.1%) and poplar (29.0%), as was expected since all three belong to the *Malpighiales *order and share lineage specific genes (Additional file 3: Table S3). Also, putative gene orthologues encoding proteins from a broad diversity of taxa (247 genera, in total) have been found, including mostly unnamed proteins from *Vitis *(15.3%), *Arabidopsis *(7.5%), rice (1.8%), sorghum (0.8%) and maize (0.4%) (Additional file 3: Table S3). Protein families such as cytochrome P450 (159 BES), kinase (378 BES) and proteins associated with disease resistance including rust resistance (108 BES) were relatively abundant (Additional file 3: Table S3). Around 127 BES were found to harbour genes or gene families encoding proteins involved in pathways associated with oil metabolism, mostly orthologous to *Ricinus communis *and *Populus trichocarpa *(Additional file 4: Table S4).

**Figure 3:**
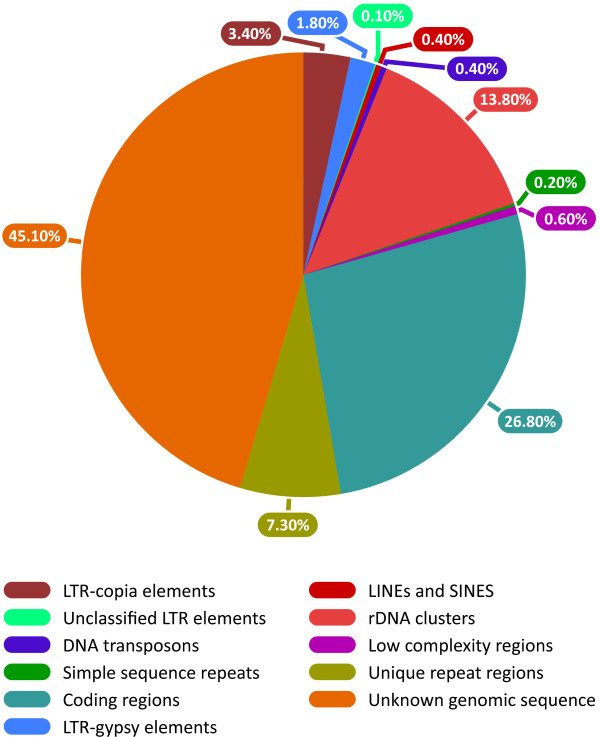
**Estimates of the composition of the flax genome (*Linum usitatissimum *cv CDC Bethune) based on BES analyses**.

A summary of the flax genome composition based on the BES annotation is presented in Figure 3. Overall, known fractions account for ~54.9% of the genome. Interspersed repeat and SSR fractions occupy ~20.7%, comprising LTR-*copia *elements (3.4%), LTR-*gypsy *elements (1.8%), LINES and SINES (0.4%), unclassified (0.1%), DNA transposons (0.4%), rDNA sequences (13.8%), SSRs (0.2%) and homopolymer tracks (0.6%). Coding regions account for 26.8% and the potential uncharacterized repeat region of the genome occupies ~7.4%. The unknown genomic sequence occupies ~45.1% of BES data and thus, as a sample, represents the estimate for the whole genome.

### Gene ontology

**Figure 4:**
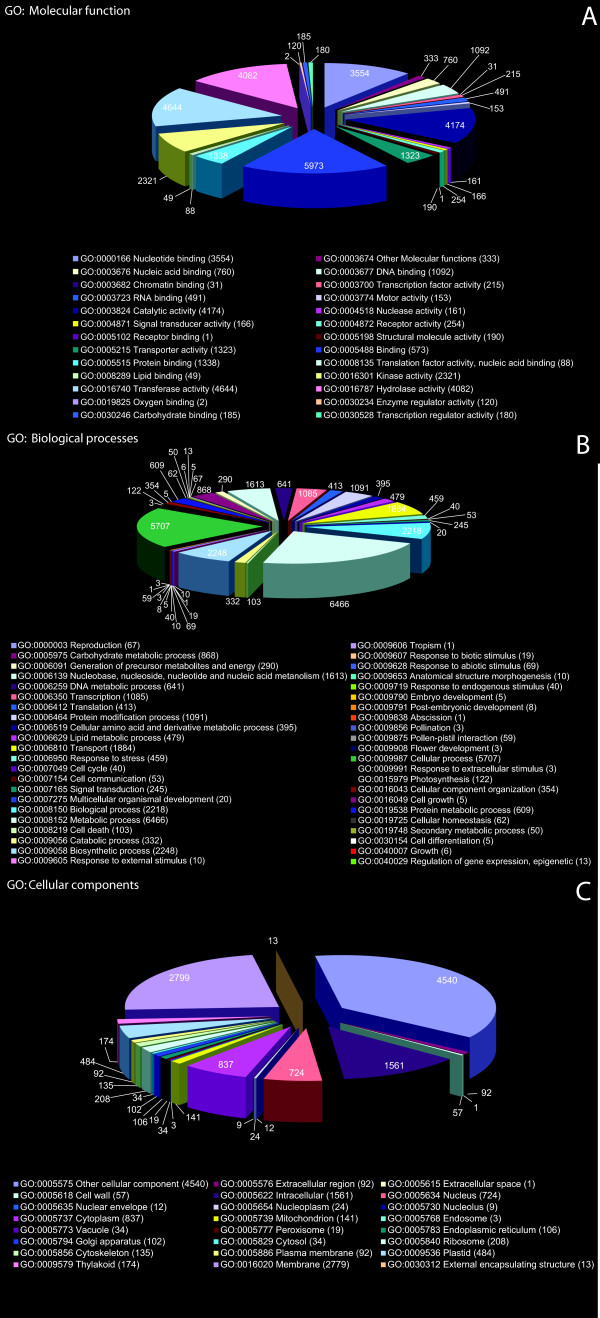
**Distribution of GO-slim annotations of gene products predicted from BAC-End sequences: **A. Molecular functions; B. Biological processes and C. Cellular locations.

Mapping of predicted proteins from 24,962 BES to the UniProt database yielded 45,380 GO annotations as a result of multiple associations of individual predicted proteins with multiple functions, processes or components (Additional file 5: Table S5). Corresponding plant GO-slim categories were obtained for all three independent GO components namely, molecular functions (Figure 4A; Additional file 6: Table S6), biological processes (Figure 4B; Additional file 7: Table S7) and cellular components (Figure 4C; Additional file 8: Table S8). The top four GO categories for molecular function were 'binding' (19%), 'transferase activity' (15%), 'catalytic activity' (13%) and 'hydrolase activity' (13%). Similarly, in the categorization of biological processes, protein signatures associated with 'metabolic processes' (23%), 'cellular processes' (20%) and 'biosynthetic processes' (8%) were predominant among annotations representing 44 processes in total. Approximately 1.7% of the catalogued proteins were assigned with roles in lipid metabolism/catabolism.

## Discussion

### Quality of the contig assembly

We report here the first genome-wide physical map of flax, generated using large insert BAC clones. Factors such as number of restriction enzymes used in the library construction, source clone genome coverage and the statistical parameters 'tolerance' and 'Sulston cutoff score' determine the quality of the map [13]. The use of two restriction enzymes minimises the proportion of underrepresented genomic regions due to non-uniform distribution of restriction sites [57]. It was reported that contig length increased rapidly when the haploid genome representation of source clones increased from 5X to 10X [58]. In the present study, BAC libraries from the cultivar CDC Bethune constructed with two restriction enzymes, namely *HindIII *and *BamHI*, were used and the source clones used to construct the physical map were estimated to have haploid genome coverage of ~10X. High stringency parameters (low tolerance value and low Sulston score value stipulating long clone overlaps) employed in our study would have minimized false positives and ensured high accuracy of contig assemblies, as reported in other plant genomes such as *Arabidopsis *[19], rice [16], apple [59] and poplar [60]. Map quality is also inferred from the number of contigs [58] and maps with fewer large contigs obtained with high stringency parameters, as observed in the present study, can be considered robust.

### Genome coverage of the assembly

The map generated in this study which consists of 416 contigs spanning ~368 Mb, represents ~54.5% of the haploid genome when using the original size estimate of 675 Mb ([5]; http://data.kew.org/cvalues/). However, if compared against the revised genome size estimate based on CDC Bethune (370 Mb; Michael Deyholos and David Galbraith, personal communication), the coverage of the genomic regions by the physical map would represent ~99.4%. Considering that ~13.8% of flax genome is assessed to contain rDNA sequences (BES based estimate; present study) whose fingerprints were removed at the editing stage (with the exception of contig 3 and part of contig 52), upstream to the assembly process, the current genome-wide physical map could be considered comprehensive.

The gaps in the contigs represent repetitive portions such as the nucleolar organizer region (NOR) and centromeres which were filtered out during the editing stage because of their highly identical fingerprints or because they represent fractions of the genome devoid of restriction sites for the enzymes used in library construction [13]. Gaps may also arise due to collapse of recently duplicated segments [61]. Physical maps of poplar [60], wheat 3B [62] and grapevine [63] were found to have 80%, 82% and 72% haploid genome coverages, respectively. On the other hand, due to either underestimation of actual genome sizes or the inability to detect potential overlaps among contigs, more than 1X coverage of the actual genome sizes by physical maps were reported for rice (1.05X; [16]), soybean (1.26X; [21]), apple (1.24X; [59]), Brassica (1.3X; [23]) and *Brachypodium *(1.38X; [24]). This being the first reported physical map of the flax genome, it provides a frame work for accessing specific target regions harbouring loci with economic/biological importance for marker development and positional cloning using large insert BAC clones.

### Anchoring contigs to the genetic map

A physical map orders genomic regions based on clone overlap whereas a linkage map positions markers based on recombination breakpoints [20]. Anchoring of contigs to a genetic map through shared markers validates the assembly and provides access to specific genomic regions for fine mapping and map based cloning of target genes/QTLs. Out of 96 contigs anchored with SSR markers, 60 contigs could be unambiguously assigned to genomic regions, since multiple positive clones identified with a single marker or set of markers were assembled to an individual/unique contig. Similarly, analyses of contigs having two or more markers indicated that genetically linked markers from 12 of the 24 published linkage groups of flax [47] were included in the same contigs, further validating the accuracy of the assembly. However, conflicts in positioning of 18 markers into more than one contig (for example, marker Lu361 mapping to four different contigs), could represent either paralogous copies of genes or duplicated segments as reported in soybean [21]. In other words, the presence of 36 contigs with conflicting marker positions may suggest the possibility that flax could be a diploidized ancient polyploid, since paleopolyploidy is ubiquitous among angiosperms [64]. Such ambiguities could be investigated further by anchoring the contigs with more markers that are genetically mapped so that unidentified overlaps between contigs could be unearthed. As well, the addition of more markers to contigs could anchor the contigs lacking markers to their respective positions across the genome. For instance, 1704 markers were employed to integrate 284 contigs with 12 linkage groups of the rice genome [16]. The current map will, moreover, serve as a scaffold to assist in the assembly of the whole genome shotgun sequence [65].

### Mobile genetic elements

**Figure 5:**
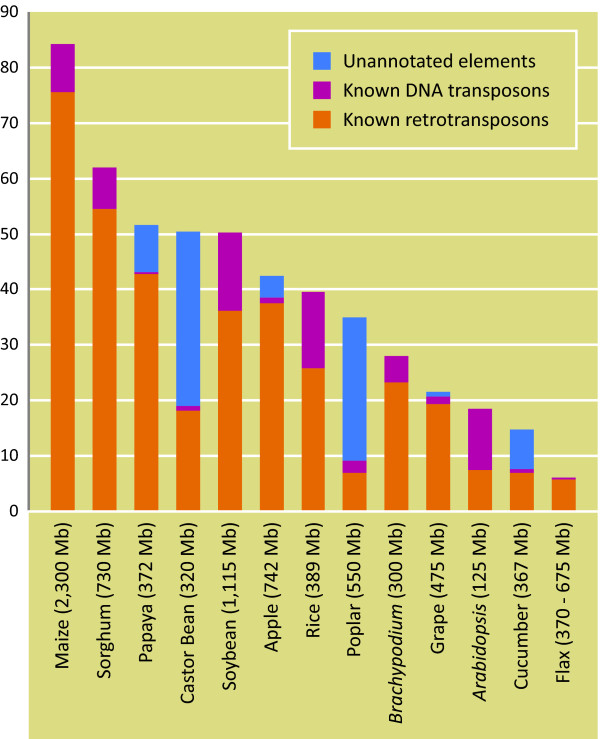
**Transposable element (TE) composition in sequenced plant genomes in comparison with the BES-based estimates in flax**. The data regarding the TE composition of other plant genomes were taken from [74; papaya] [45; castor bean] [80; apple and other genomes]. Please refer to Additional file 9: Table S9 for more details.

Transposable elements play significant roles in the evolution of structure, function and regulation of expression of genes and genomes [66,67]. Mobile DNA also significantly impacts the genome size [68]. Among various repeat prediction tools, Repeatmasker is widely used for identifying repeats in genomes [69] using Repbase, a manually curated high quality database of consensus sequences of eukaryotic repeat elements [49]. Repeatmasker analysis identified ~6.1% of the BES of flax as having homology to known transposable elements. This estimate of known mobile genetic elements is the lowest among twelve plant genomes whose whole genome sequences are available to date, namely *Arabidopsis *(14%, [70]), rice (34.7%, [71]), poplar (35%, [72]), grapevine (21.5%, [73]), papaya (51.9%, [74]), sorghum (62%, [75]), maize (84.2%, [76]), cucumber (14.8%, [77]), soybean (50.3%, [78]), *Brachypodium *(28.1%, [79]), castor bean (50.3%, [45]) and apple (42.4%, [80]) (Figure 5; Additional file 9: Table S9). However, the unknown portion of the flax genome, including the novel repeat fraction of the genome (Figure 3), would be a reservoir of new mobile genetic elements and hence the proportion of transposable elements in flax is predicted to increase with the characterization of this currently unknown fraction. In castor bean and poplar, ~31.3% and 25.9% of the genome were represented by unannotated/unknown elements [45,72]. The proportions of known retrotransposons in flax were predominant over DNA elements, as reported in other plant genomes, with the exception of *Arabidopsis *(Additional file 9: Table S9). However, flax was found to have a higher proportion of *copia *retrotransposons than *gypsy *elements compared to all other sequenced plant genomes where *gypsy *elements predominated (Additional file 9: Table S9), indicating the possibility of uncharacterized sequences as a warehouse of new members which may alter the proportion of *copia/gypsy *elements. Recently, the repetitive portion of the banana genome was found to harbour a higher proportion (16%) of *copia *elements than *gypsy *elements (7%) [81]. Only 62 known families of transposable elements have been identified from the BES, far fewer than the whole genome based estimates of 1323 families in maize, 300 families in rice and 510 families in soybean [82].

Among the known flax transposable elements, *dLUTE*, FL2, FL9 and FL11 were not found to have any matches in the BES dataset. Interestingly, the partial sequence of the element FL4 (GenBank ID GU929874) was found to have 365 hits, representing a copy number estimate of ~516 in the genome when taking into consideration the genome size, size of the BES database and redundancy of the library. This retroelement may serve for developing a retroelement based marker system, exploiting the polymorphism created at their insertion sites which are useful in diversity analysis as a fingerprinting tool, as shown in a recent study characterizing the genetic structure of flax germplasm [83].

### Ribosomal DNA (rDNA) sequence

Ribosomal RNA genes as a component of ribosomes are a predominant class of housekeeping genes. Based on Repeatmasker analysis, rDNA was found to occupy ~13.8% of the total length of the BES, accounting for ~7.5 Mb from 13,228 reads. Independent validation by BLASTn searches against the TIGR repeat database yielded a similar rDNA estimate of 13.5%, as the proportion of total length of BES from 13,746 reads. BES from 13,166 clones matched rDNA homologues from both Repbase and TIGR plant repeat databases, indicating their significant presence in the flax genome. This estimate of rDNA content in flax was found to be much higher compared to BES based estimates in *Brassica rapa *(2.5%; [32]), carrot (2.06%; [40]), *Brachypodium *(1.2%; [36]), *Musa acuminata *(1.12%; [81]), maize (0.82%; [29]) and Wheat 3B (0%; [33]). However, the choice of the restriction enzymes used in the construction of large insert libraries was found to have an influence on the estimates, as reported in tomato in which the rDNA estimates were 0.04%, 2.98% and 8.58%, respectively based on libraries constructed with *HindIII*, *EcoRI *and *MboI *[37]. Similarly in potato, the *HindIII *library based estimate (0.03%) was found to be lower than the *EcoRI *library based estimate of 0.53% [37]. Recent studies indicated that rRNA genes are silenced by epigenetic means for dosage control and thereby their copy number may not represent their abundance in the transcriptome [84].

Detailed annotation obtained from flax BES having significant similarity to the entries in the TIGR plant repeat database indicated that 13,258 BAC-end reads harbour 45S rRNA and 407 reads harbour 5S rRNA. Though cytogenetic studies identified two nucleolar organizer regions harbouring 45s rDNA loci with tandem arrays of repeating units [8], our study raises the possibility that that there are more than two NORs or that the two NORs contain an unprecedented large number of copies of 45S rRNA repeat units per locus. A study of 45 *Brassicaceae *species indicated multiple 45S rDNA sites across the genome, as observed in *Brassica rapa *(10 sites) and *Brassica juncea *and *Brassica napus *(12-14 sites each) [85]. Comparatively fewer 5S rDNA would have been detected due to differences in the number of restriction sites because flax 45S rDNA was found to have restriction sites for both *HindIII *and *BamHI *whereas 5S rDNA has a site for *BamHI *only (data not shown). Moreover, extensive methylation of 5S rDNA resulting in incomplete restriction digestion [8,9], a higher degree of sequence variation observed among 5S rDNA classes [10] and a smaller length of repeat units (350 bp-5S rDNA vs 8.6 kb-45S rDNA) combined with factors such as partial digestion by a hexanucleotide cutter with a probability of finding a restriction site per ~4096 bp and size selection during library construction would have contributed to reduced sampling of 5S rDNA sequences among the BES. Interestingly, their high copy number was reported to facilitate genome integrity by favouring sister chromatid cohesion during recombination repair in yeast [86], a hypothesis that could provide some insights into the genome size variations of the genotrophs.

### Novel repetitive sequences

Approximately 7.4% of the BES were found to be novel repetitive sequences not available in public sequence databases. This estimate was analogous to BES-based estimate of novel repeats in carrot (8.4%; [40]) and *Brachypodium *(7.4%; [36]) and is likely due to the rapid evolution of repetitive sequences which have comparatively fewer constraints than coding regions [87]. When validated for their unique nature with longer queries after assembly, by comparison against various databases, including plant repeat databases, ~28.9% of the sequences were homologous to flax LTR retrotransposons such as FL4, FL6 and FL7. These LTR retrotransposons were not amenable for detection while repeat masking, because of the high degree of divergence possible in LTR domains, as reported in *Brachypodium *[36]. Sequences similar to *Linum *microsatellite sequences, multi-gene families such as 5S rRNA, flax rust resistance protein families and pectin methylesterase (*pme3*) were also observed. Interestingly, three of the novel flax specific repeats were similar to the *Linum *insertion sequence characterized in genotrophs induced by the environment [7] constituting the first report of the presence of *Linum *insertion sequence in the cultivar CDC Bethune. The novel repetitive fraction may represent new flax-specific transposable elements and needs further characterization.

### Simple sequence repeats (SSRs)

BES are found to be a good source of SSRs, a class of markers widely used in generating linkage maps, to scan the genome for specific loci associated with agronomically important complex traits [88]. In our study, 4064 putative SSRs markers have been identified from ~54.6 Mb of BES, giving a density of one SSR per every ~13.4 kb of the flax genome, compared to an earlier study of mining SSRs from ESTs in which one SSR per 16.5 kb was reported [89]. The estimates of ~38.7% dinucleotide and 53.7% trinucleotide repeats in SSRs from BES are different from the EST derived estimates where trinucleotide repeats (76.9%) were more abundant than dinucleotide repeats (13.9%). The polymorphism of these BES-SSRs is currently being assessed and polymorphic SSRs will be integrated with the first SSR based flax genetic map [47] and to anchor the physical and genetic maps. Comparative analysis of SSR motif classes and composition among sequenced plant genomes *vis-à-vis *flax indicated predominance of dinucleotide repeats in all genomes with the exception of *Brachypodium *and flax (Figure 2; Additional file 2: Table S2). The motif (AT/AT)_n _was found to be predominant in 11 of 13 genomes whereas in maize and *Brachypodium*, (CT/AG)_n _was predominant. Similarly, among trinucleotide motifs, (AAT/ATT)_n _was found to be predominant in six genomes (Additional file 2: Table S2), whereas in flax and *Arabidopsis *(GAA/TTC)_n _was the major component. However, the whole genome sequence of flax would provide a more comprehensive characterization of flax SSR motifs that may alter the abundance and composition of motifs inferred from BES.

### Coding regions and gene content

Sequence based similarity searching has been widely used for computational identification of genes and assignment of putative functions by querying public databases [90]. In our present study, 26.8% of BAC-end reads were found to have similarity with transcripts from flax itself and only 21.1% of reads matched to NCBI-ESTs, in spite of manyfold differences in the sizes of these two databases. This result suggested that a portion estimated at 5.6% of the flax transcriptome is unique in its nature, representing flax-specific genes. Indeed, a still higher proportion of flax specific genes was reported from a recent study where only 21.3% to 62.9% of 59,626 EST-derived unigenes were found to have similarity to known genes from other genomes [12]. The cumulative match length identified ~9.6% and ~6.1% as the proportions of open reading frames, based on matches to flax-ESTs and NCBI-ESTs, respectively in the same range as grapevine exons-CDS (6.9%; [73]). The absence of introns in the ESTs, a higher level of conservation expected at protein level and use of a different mining criterion, resulted in an increased proportion of reads (30.9%) having significant hits against the *nr *protein database (cut off E = e^-5^), though this only represents the similarity to known proteins in other organisms. As expected, at a further increased threshold level (E = e^-25^), the proportion of clones with potential coding regions decreased (17.7% of the total BES), but remained comparatively higher than the BES-based assessment of coding regions in carrot (10%; [40]), apple (8.6%; [39]), *Musa acuminata *(11%; [34]), *Brassica rapa *(11%; [32]), and comparable to or lower than the coding fractions reported in papaya (19.1%; [31]), white clover (24.9%; [35]), common bean (29.3%, [22]), *Brachypodium *(25.3%; [36]), citrus (36.0%; [38]). A total of 11,180 BES (13.8%) shared evidence for transcribed coding regions as they produced hits from both EST and nr-protein databases.

Assuming a median gene size of 3.4 kb reported in the grapevine genome [73] and using our estimate of transcribed portion (26.8%) having evidence based on flax-ESTs, we could predict from 29,164 to 53,245 genes corresponding to genome size estimates of 370 Mb (Michael Deyholos and David Galbraith, personal communication) to 675 Mb [5], respectively. The lower end of the range is comparable to the predicted number in castor bean (31,237 genes; [45]) and the higher end is comparable to the number in apple (57,386 genes; [80]), the highest among twelve plant genomes sequenced so far. In apple, with a possibly comparable genome size species (742 Mb), genome-wide duplication was reported as the cause for the large number of genes. The high proportion (~50%) of low copy sequences in flax [7] and relatively high gene content could also result from an ancient polyploidization event which suggests that the repertoire of genes in flax may potentially harbour duplicate genes as paralogous copies or gene families. Whole genome sequence analysis indicated that ancient polyploidization was a typical feature of angiosperms namely, *Arabidopsis *[70], poplar [72], sorghum [75], maize [76], castor bean [45] and soybean [78].

### Gene ontology

Distribution of predicted protein sequences from BES to high-level GO terms suggested the presence of a broad range of categories from all GO-slim functional classes (Figure 4A). Since predicted proteins can be assigned to more than one functional category, there were more annotations (31,880) than total proteins (24,962), as reported even in a simple eukaryote such as yeast [44]. Proteins with 'binding' domains are overrepresented followed by other domains such as 'catalytic activity', similar to GO categorization of flax unigenes reported recently [12], because of the conservation of basic biological processes across eukaryotes. Also, protein signatures associated with ~44 biological processes have been identified; including 479 (1.7%) annotations assigned a role in lipid metabolic processes from ~127 BES. The cytochrome P450 superfamily associated with synthesis of secondary metabolites, as well as the kinase family of proteins including serine/threonine receptor kinase with roles in disease resistance were relatively overrepresented, as reported in tomato and potato [37].

## Conclusions

A total of 43,776 BAC clones from the library of the flax cultivar CDC Bethune was used to construct the first genome-wide physical map and to generate BES, annotation of which unearthed the uniqueness of the flax genome. The physical map assembled from 32,025 high quality fingerprints consists of 416 contigs spanning ~368 Mb, roughly 54.5% to 99.4% of the estimated genome sizes. The N50 size of the contigs was estimated to be ~1,494 kb and the longest contig was ~5,562 kb. As a genomic resource, this map will be useful for fine mapping of target genomic regions and map-based cloning of genes/QTLs. Also, generation and annotation of BES, totalling 54.6 Mb (~8-14.8% of the haploid genome) suggested that known repetitive fractions and coding fractions account for ~28.1% and 26.8% of the genome, respectively. Among the known repetitive fractions, ribosomal DNA accounts for ~13.8%, the highest proportion reported so far in plant genomes. In contrast, the flax genome was found to have a smaller proportion of known transposable elements (~6.1%) than published plant genomes. BLASTn searches against an in-house flax-EST database (db) and the NCBI-EST db found 26.7% and 21.1% homology, respectively, suggesting that approximately 5.6% of the coding region is unique in flax. As expected, BLASTx predicted flax proteins were similar to hypothetical proteins from castor bean (33.1%) and poplar (29.0%) because of their shared lineage (*Malpighiales*). Gene ontology (GO) terms associated with molecular function, biological processes and cellular components indicated the presence of a broad range of catalogued proteins and ~1.7% of predicted proteins were assigned roles in lipid biosynthetic and catabolic processes. Analysis of the BES has provided initial insights into the uniqueness of the flax genome among other characterized plant genomes. Both the physical contigs and paired-end reads from large insert BAC clones, will be helpful to validate the accuracy and reliability of the whole genome shotgun sequence assembly of flax.

## Authors' contributions

SC designed the study, generated data, participated in the analysis and interpretation of data and co-wrote the manuscript; RRag carried out the analysis, interpretation of data and co-wrote the manuscript. RRat participated in the analysis and interpretation of data. All the authors read and approved the final manuscript.

## Supplementary Material

Additional file 1 **Table S1: Physical map of flax *Linum usitatissimum *L. cultivar CDC Bethune**. The details of the FPC contigs are provided in this spreadsheet. The table columns detail, from left to right: contig number, length of contigs (in Consensus Band-CB units), estimated physical length (bp), number of buried clones in a given contig, total number of clones and number of integrated genetic markers in a given contig. Click here for file

Additional file 2 **Table S2: SSR abundance and composition of sequenced plant genomes and flax BES**. Data regarding SSRs mined from whole genome assemblies of apple, soybean, maize, grapevine, poplar, papaya, sorghum, rice, castor bean, cucumber, *Brachypodium distachyon*, *Arabidopsis thaliana *and the BES of flax are given in this spreadsheet. The table columns detail, from left to right: plant genome name, genome size (Mb), total size of examined sequences (bp), total number of identified motifs, number of dinucleotide motifs, number of trinucleotide motifs, number of tetranucleotide motifs, number of other motifs, the predominant dinucleotide motif, the predominant trinucleotide motif, the predominant tetranucleotide motif and the reference. Click here for file

Additional file 3 **Table S3: Summary of putative protein hits based on BLASTx homology searches of BES against the nr database**. Click here for file

Additional file 4 **Table S4: List of BES associated with oil metabolism deduced from BLASTx homology searches**. Click here for file

Additional file 5 **Table S5: Summary of mapping of putative proteins from BLASTx homology searches against UniProt entries for GO terms**. Click here for file

Additional file 6 **Table S6: Distribution of GO annotation of functional classes of gene products encoded from BAC-End sequences**. Click here for file

Additional file 7 **Table S7: Distribution of GO annotation of biological processes associated with gene products from BAC-End sequences**. Click here for file

Additional file 8 **Table S8: Distribution of GO annotation of cellular locations of gene products from BAC-End sequences**. Click here for file

Additional file 9 **Table S9: Mobile genetic elements content in the sequenced plant genomes and flax BES**. Click here for file
